# Development of the Happy Hands Self-Management App for People with Hand Osteoarthritis: Feasibility Study

**DOI:** 10.2196/59016

**Published:** 2024-10-29

**Authors:** Anne Therese Tveter, Cecilie Varsi, Marit Kristin Maarnes, Stein Jarle Pedersen, Barbara S Christensen, Thale Beate Blanck, Sissel B Nyheim, Tim Pelle, Ingvild Kjeken

**Affiliations:** 1 Health Service Research and Innovation Unit Center for Treatment of Rheumatic and Musculoskeletal Diseases (REMEDY) Diakonhjemmet Hospital Oslo Norway; 2 Faculty of Health and Social Sciences University of South-Eastern Norway Drammen Norway; 3 Unit for Clinical Activity Division of Medical Services Diakonhjemmet Hospital Oslo Norway; 4 Faculty of Medicine University of Oslo Oslo Norway; 5 Division of Rheumatology and Research Diakonhjemmet Hospital Oslo Norway; 6 Norwegian Rheumatism Association Oslo Norway; 7 Department of Primary and Community Care Radboud University Medical Center Nijmegen Netherlands

**Keywords:** Osteoarthritis, hand exercises, home exercise, first-line treatment, mHealth, eHealth, self-management, app development, design, usefulness, satisfaction, activity performance, social cognitive theory, behavior change

## Abstract

**Background:**

Patient education, hand exercises, and the use of assistive devices are recommended as first-line treatments for individuals with hand osteoarthritis (OA). However, the quality of care services for this patient group is suboptimal in primary care.

**Objective:**

The overarching goal was to develop and evaluate feasibility of an app-based self-management intervention for people with hand OA. This feasibility study aims to assess self-reported usability and satisfaction, change in outcomes and quality-of-care, exercise adherence and patients’ experiences using the app.

**Methods:**

The development and feasibility testing followed the first 2 phases of the Medical Research Council framework for the development and evaluation of complex interventions and were conducted in close collaboration with patient research partners (PRPs). A 3-month pre-post mixed methods design was used to evaluate feasibility. Men and women over 40 years of age diagnosed with painful, symptomatic hand OA were recruited. Usability was assessed using the System Usability Scale (0-100), while satisfaction, usefulness, pain, and stiffness were evaluated using a numeric rating scale (NRS score from 0 to 10). The activity performance of the hand was measured using the Measure of Activity Performance of the Hand (MAP-Hand) (1-4), grip strength was assessed with a Jamar dynamometer (kg), and self-reported quality of care was evaluated using the Osteoarthritis Quality Indicator questionnaire (0-100). Participants were deemed adherent if they completed at least 2 exercise sessions per week for a minimum of 8 weeks. Focus groups were conducted to explore participants’ experiences using the app. Changes were analyzed using a paired sample t test (mean change and 95% CI), with the significance level set at *P*<.05.

**Results:**

The first version of the Happy Hands app was developed based on the needs and requirements of the PRPs, evidence-based treatment recommendations, and the experiences of individuals living with hand OA. The app was designed to guide participants through a series of informational videos, exercise videos, questionnaires, quizzes, and customized feedback over a 3-month period. The feasibility study included 71 participants (mean age 64 years, SD 8; n=61, 86%, women), of whom 57 (80%) completed the assessment after 3 months. Usability (mean 91.5 points, SD 9.2 points), usefulness (median 8, IQR 7-10), and satisfaction (median 8, IQR 7-10) were high. Significant improvements were observed in self-reported quality of care (36.4 points, 95% CI 29.7-43.1, *P*<.001), grip strength (right: 2.9 kg, 95% CI 1.7-4.1; left: 3.2 kg, 95% CI 1.9-4.6, *P*<.001), activity performance (0.18 points, 95% CI 0.11-0.25, *P*<.001), pain (1.7 points, 95% CI 1.2-2.2, *P*<.001), and stiffness (1.9 points, 95% CI 1.3-2.4, *P*=.001) after 3 months. Of the 71 participants, 53 (75%) were adherent to the exercise program. The focus groups supported these results and led to the implementation of several enhancements in the second version of the app.

**Conclusions:**

The app-based self-management intervention was deemed highly usable and useful by patients. The results further indicated that the intervention may improve quality of care, grip strength, activity performance, pain, and stiffness. However, definitive conclusions need to be confirmed in a powered randomized controlled trial.

**Trial Registration:**

NCT05150171

## Introduction

Hand osteoarthritis (OA) is a highly prevalent rheumatic joint disease [1], and the number of individuals affected by debilitating hand OA is expected to increase in the coming decades due to rising life expectancy [2]. The lifetime risk of developing hand OA is 50% for women and 25% for men, with older age being a significant risk factor [3]. Today, OA is recognized as a disease characterized by chronic abnormal remodeling, affecting the entire synovial joint. The resulting structural and functional failures negatively impact body functions and structures, activity performance, work ability, and health-related quality of life. The functional impairment caused by hand OA is often comparable to that of rheumatoid arthritis (RA), but currently, there are fewer established and available treatment options [4]. There is currently no cure or disease-modifying drug for OA. Based on updated evidence, the 2018 European Alliance of Associations for Rheumatology (EULAR) recommends a stepped-care approach, with patient education, hand exercises, and assistive devices as first-line treatments for hand OA [2]. Orthoses are recommended for patients with thumb base OA (a subgroup of hand OA). Nonsteroidal anti-inflammatory drugs can be used for short-term symptom relief, while surgery should only be considered when other treatment options have failed [2]. However, a Norwegian trial [5] found that only 21% of patients with thumb base OA had received the recommended nonpharmacological treatments before being referred by their general practitioner (GP) for surgical consultation [6]. Several efforts have been made to develop models for delivering recommended OA care, but to date, these are primarily available for individuals with hip or knee OA [7-9], although some have focused on hand OA [10,11]. People with hand OA often report encountering health professionals who either dismiss their symptoms or suggest that hand OA is a natural consequence of aging, with no available interventions or treatments [12,13]. Recent research supports the notion that the overall quality of care for this patient group is suboptimal [14]. Therefore, models for hand OA care that provide evidence-based treatment to all patients are urgently needed.

Self-management plays a crucial role in health care, empowering people with chronic diseases to monitor their condition and implement lifestyle changes necessary to maintain a satisfactory quality of life [15]. It is often defined as “the ability of individuals to manage symptoms, treatment, lifestyle changes, and the psychosocial and cultural consequences of health conditions” [16]. In rheumatology care, self-management support has been provided through group programs since the 1980s and increasingly as part of routine clinical practice, primarily through face-to-face sessions [17]. Recently, studies have examined digitally delivered programs for hip and knee OA [18,19]. When designing self-management interventions, it is important to include strategies that facilitate and promote behavior changes, such as goal setting, self-monitoring, feedback and reinforcement, social support, problem-solving, and stepwise progression [20]. Furthermore, the complexity of modern medicine, coupled with the growing use of digital health information, places greater demands on individuals’ health literacy [21]. Health literacy refers to people’s ability to access, understand, evaluate, and use information and health services to promote and maintain their own health and well-being, as well as that of others around them. It has been particularly challenging for older adults, individuals with chronic diseases, those with lower education levels, and those with limited language proficiency [22]. Inadequate health literacy is linked to difficulties in understanding health information, limited knowledge of diseases, and lower medication adherence, all of which can contribute to poor health outcomes, insufficient or ineffective use of health care services, and health disparities [21]. Therefore, it is crucial to involve the target population in the development of the content, format, and design of self-management interventions [23].

Strategic documents emphasize the use of eHealth in self-management and the more efficient utilization of health care resources [24]. One potential model for OA care involves the digital delivery of self-management programs, particularly through smartphone technology (ie, mobile health [mHealth]). The development and use of mHealth devices and apps signify a paradigm shift in health care [25], a shift that has been further accelerated by the COVID-19 pandemic. These technologies can serve multiple functions, including delivering care, facilitating adherence, collecting data, and enabling self-monitoring. mHealth technology also allows for more flexible delivery and empowers patients to take greater responsibility for their health and treatment decisions. Several reviews have concluded that mHealth apps are effective in delivering health interventions across various domains and health conditions [26-28]. Recently, the EULAR has proposed considerations for remote care [29], as well as for the development, evaluation, and implementation of mHealth apps [25] for individuals with rheumatic and musculoskeletal diseases. In a systematic review from 2019, the authors were unable to identify any studies describing the development of evidence-based mHealth apps focused on OA or evaluating the effectiveness of such apps in OA management [30]. However, more recent efforts have been made to develop and test mHealth interventions for patients with hip and knee OA through the dr. Bart app [31,32] and Joint Academy [33]. Studies of these interventions have demonstrated effects on pain and functional measures compared with usual care [34], as well as a substantial decrease in the economic burden of OA for patients and health care services [31,35]. However, at the start of our study, no published data were available on the use of an mHealth approach to delivering evidence-based interventions to patients with hand OA. Therefore, it is worthwhile to explore the potential of mHealth in managing hand OA.

The overarching goal of this study was to develop and test the feasibility of an app-based self-management intervention for individuals with hand OA—the Happy Hands app. The specific aims of the feasibility study were to assess patient-reported usefulness and satisfaction with the intervention, changes in pain, stiffness, activity performance of the hand, grip strength, self-reported quality of care, and adherence to the app’s content. Additionally, we aimed to explore patients’ experiences using the app.

## Methods

### Overview

In this study, we used the updated Medical Research Council framework for the development and evaluation of complex interventions [36]. The Medical Research Council framework outlines 4 phases, of which this study addresses the first 2: (1) intervention development or identification and (2) feasibility assessment of the intervention and evaluation design. The remaining phases are (3) intervention evaluation and (4) impactful implementation. Throughout all phases, it is crucial to account for contextual factors, develop and refine the program theory, engage all relevant stakeholders, identify key areas of uncertainty, refine the intervention based on feedback, and consider economic aspects [36]. The app development and feasibility assessment were conducted through iterative processes.

### Intervention Development

The overarching goal of developing the app was to create a standalone intervention that supports and empowers individuals with hand OA to self-manage their condition, regardless of their location, by providing access to information about recommended treatments and guidance on hand exercises.

The intervention development phase began with a literature review focusing on patient experiences, exercise programs, and treatment recommendations for individuals with hand OA. This review revealed studies discussing the impact of and patients’ experiences living with hand OA [17,37-41], hand exercise programs for hand OA [42-44], and updated evidence-based treatment recommendations [2,15,45]. To identify user needs and requirements, 2 female patient research partners (PRPs), TBB and SBN, who have hand OA and extensive experience working with researchers, were interviewed by the principal investigators, ATT and IK, to discuss the app’s content and design. This needs assessment also incorporated valuable insights from previously conducted patient interviews, which significantly contributed to the creation of an informative leaflet aimed at guiding patients on effective strategies for managing everyday life with hand OA [46]. The PRPs reported a wide range of everyday challenges, self-management strategies, and preferences for app design and content. Their needs and requirements are summarized in Textboxes 1 and 2.

The content and design of the app were subsequently discussed within the project group, which included 2 occupational therapists, a physiotherapist, a nurse, 2 doctors (all working as clinicians or researchers), and the 2 aforementioned PRPs.

Social Cognitive Theory was identified as a suitable theoretical framework to guide the development of the app, emphasizing that behavior change occurs through perceived self-efficacy; perceived benefits or expectations that a behavior will lead to a positive outcome; and perceived control, which is the belief that specific behaviors can be shaped and influenced [20]. This theory informed the selection of videos and other elements to include, as well as the content and wording within the videos.

To ensure that we addressed various elements of behavioral change, we utilized the behavior change technique (BCT) Taxonomy (version 1) to map the elements in the app [47]. For example, the rewards included in the app were categorized as “Rewards and threats” within the BCT taxonomy. Because of the ongoing commercialization of the app, we refrain from providing detailed reports on its content. However, the app includes elements that were mapped to 11 of the 16 taxonomy cluster labels. Based on feedback from the PRPs, we emphasized that all included BCTs should be positively directed. Furthermore, we recognized that participants may respond to different behavioral change techniques, and thus aimed to incorporate a diverse range of techniques to engage as many participants as possible.

**The prioritized themes and subthemes provided by the patient research partners in the development process of the app. Subthemes included in the app are in italics.**

1. Information about hand osteoarthritis (OA)
  - The anatomy of the hand
  - What is hand OA and why do people get it?
  - Symptoms and prognosis of hand OA
  - Recommended treatment for hand OA
2. Care and communication
  - When to seek care
  - How to prepare for a visit to the doctor
  - When to seek help in specialist health care
  - Support from health care professionals
  - Support from peers
3. Motivation and self-management
  - Goal setting
  - Making plans
  - Motivation for self-management
  - Managing everyday life
  - Healthy eating
4. Hand exercises
  - Why exercise?
  - How to exercise?
  - Exercise program
  - Individualization and progression of exercise programs
5. Assistive devices and ergonomic working methods
  - Ergonomic principles and working methods
  - Use of assistive devices
  - Practical advice from people with hand OA
6. Orthoses
  - Why and when to use orthoses?
  - Which orthoses to use for what?
  - Where to get orthoses

**Important aspects indicated by the patient research partners in the development process of the app.**

1. Manageable
  The app must be easy to use and not overly time-consuming, meaning the intervention should take a maximum of 25 minutes per day, 3 days per week. The preferred format for presenting the content is videos with soundtracks and the option to add text. Each video should be relatively short, with a duration of no more than 3-4 minutes.
2. Motivation and empowerment
  The app must be motivating and encouraging. Information should be easy to understand, avoiding descriptions of “worst-case scenarios” and any frightening illustrations. Users should receive feedback on their success and progress to boost motivation and self-efficacy. Furthermore, all feedback should focus on the users’ positive outcomes rather than what they have not yet achieved. Quizzes should assess the users’ knowledge, and the feedback should remain positive and informative. Users must also have the opportunity to retake the quiz until all questions are answered correctly.
3. Autonomy and flexibility
  The app must be flexible, allowing each user to decide when and where to use it. Any tools needed for the exercises should be small enough to fit in a handbag. The preferred platform is a smartphone or tablet, as both support flexible use. If a choice must be made between the 2, the smartphone is preferred.
4. Trustworthiness
  The app must include both research- and experience-based information. Health professionals in the videos should introduce themselves with their name, profession, and workplace to ensure clarity for the users. Individuals demonstrating exercises, working methods, or sharing experiences should be relatable to the app users.
5. Social support
  The app must include a chat function where users can share experiences and advice, as well as team up to do exercises or try different management strategies together. Users should also have the option to seek advice and supervision from an occupational therapist or physiotherapist when needed.

The app’s design was discussed with IT developers, who began creating app illustrations and a prototype based on the descriptions and requirements provided by the project group. In designing the app, we included informative videos, exercise videos, questionnaires, quizzes, feedback messages, graphs, and awards, all based on the prioritized themes identified by the PRPs (Textbox 1). The content was produced by the principal investigators, guided by the literature review, input from the PRPs, and a leaflet [46].

To ensure that the content in the app was easy to understand, the PRPs reviewed and revised all manuscripts used as the basis for the videos and texts. Health literacy principles guided the content, format, and design of the app to promote understanding, engagement, and overall effectiveness of the intervention [23]. To improve accessibility for users, all videos included both audio and text, the app was resizable without loss of content or functionality, and complex medical terminology was avoided. To assess adherence to the app’s components, we logged which informational and exercise videos users had watched.

The prototype development was carried out in four 2-week development “sprints,” during which the IT developers successively incorporated the videos, questionnaires, quizzes, feedback messages, graphs, and awards (bronze, silver, and gold medals). After each sprint, a digital meeting was held where the developers demonstrated the app prototype, discussed adaptations and design solutions with the principal investigators, and made plans for the next sprint. The PRPs participated in 1 of these meetings, which occurred when the prototype had advanced sufficiently for them to provide feedback on all elements of the app.

The final prototype of the app included monthly questionnaires, 26 informative videos, 8 exercise videos, quizzes, and personalized feedback over 3 months. The exercise program consisted of videos focusing on warm-up, finger mobility, thumb mobility, grip strength, wrist stability, thumb stability, coordination, and stretching.

Participants received reminders encouraging them to exercise 3 times per week. When starting a session, users were provided with video demonstrations to guide them in performing the scheduled exercises. At the beginning of each week, participants also received a set of informative videos. All videos were stored within the app and could be watched multiple times.

The General Data Protection Regulation (GDPR) was followed to ensure the security and privacy of participants using the app. The University Information Technology Center (USIT) at the University of Oslo, Norway, was selected to develop the app due to its expertise in creating innovative digital solutions and smartphone apps for research purposes. Notably, USIT developed Nettskjema, a tool for designing digital questionnaires that can collect sensitive data, which was customized for data collection through the app. Additionally, they developed the Services for Sensitive Data (TSD), an advanced platform that provides secure storage and analysis capabilities for sensitive data. Nettskjema seamlessly integrates with TSD through encryption and direct data transfer. Data stored in TSD can be accessed through 2-factor authentication and are only available to the project manager and selected researchers approved by the project manager.

### Feasibility Assessment Design

In the second phase, a mixed methods feasibility study with a longitudinal pre-post design was conducted [36]. Self-reported outcomes and grip strength were collected at baseline and after a 3-month follow-up. At follow-up, focus group interviews were conducted with study participants. The main aim of the feasibility study was to explore participants’ experiences with using the app, adherence to watching information and exercise videos, and their usefulness and satisfaction. Additionally, the study assessed changes in patient-reported outcomes (pain, stiffness, activity performance of the hand, and self-reported quality of care) and grip strength. The results were used to identify the needs for app adjustments and to inform the decision on whether to proceed with a randomized controlled trial to evaluate the app’s effect.

### Ethical Considerations

The feasibility study was preregistered in ClinicalTrials.gov (NCT05150171), and approval was obtained from the Regional Committee for Medical and Health Research Ethics in South-Eastern Norway (249364) as well as from the Data Protection Officer at Diakonhjemmet Hospital (00403). All participants received both oral and written information about the study and signed a digital consent form. Data were deidentified before analyses were conducted. The data collection took place from November 2021 to June 2022, and participants were not compensated for their participation in the study.

### Participants and Settings

Men and women aged 40 years and older, diagnosed with painful and symptomatic hand OA and who possessed a smartphone, were eligible for inclusion in the study. Individuals were excluded if they had cognitive deficits, were scheduled for hand surgery within the next 3 months, did not speak or understand Norwegian, or had uncontrolled serious comorbidities. The participants were recruited from the rheumatology outpatient clinic at Diakonhjemmet Hospital in Oslo, Norway; by a GP at a medical center in Fredrikstad, Norway; through a notice on the website of the Norwegian Rheumatism Association; and through an article on hand OA in the daily press. Our target was to recruit a diverse sample from among those seeking treatment in both specialist and primary health care, as well as those with complaints who had not sought treatment in health care services. Guidelines for sample size calculation in feasibility studies are not established [48], but the sample size should be large enough to inform future RCTs, typically between 30 and 50 participants [49,50]. Therefore, we aimed to recruit approximately 15 participants from each site, resulting in a minimum of 60 participants. To gather valuable insights on the use of the app, we planned to conduct at least one focus group interview at each site, with groups consisting of 3-8 participants. The sample size was guided by information power to ensure saturation of the data material [51].

At Diakonhjemmet Hospital and the medical center, participants were informed about the study by their health care providers. If participants expressed interest, their contact details were shared with the researchers, who then reached out to them. Additionally, some participants contacted the researchers directly using the contact information provided on the Norwegian Rheumatism Association’s website and in the article published in the daily press. Eligible participants were scheduled for an appointment with members of the project group (IK, MKM, and ATT) at Diakonhjemmet Hospital, the medical center, or the premises of the Norwegian Rheumatism Association. During this appointment, they completed a digital consent form, followed by a digital questionnaire and grip strength assessment. Afterward, all participants downloaded the app to their smartphones and received instructions on how to use the app and conduct the 3-month intervention. After 3 months, participants were scheduled for a follow-up appointment at 1 of the 3 recruitment sites, where they completed digital follow-up questionnaires and had their grip strength retested. Focus groups were conducted alongside the 3-month follow-up appointment, with participants selected from those who consented to participate in the focus groups. This selection aimed to ensure a diverse sample in terms of gender, age, and recruitment sites.

### Outcomes

Demographic variables included gender, age (in years), education level (categorized as low levels of education, including elementary and high school, and higher levels of education, including bachelor’s degree and above), work status (working, on all-cause sick leave, retired, on disability pension, or unemployed), and marital status (living alone or living with someone). Participants indicated which hand they experienced symptoms in (right, left, or both) and the number of painful joints (up to 15 joints in each hand). Motivation for exercising was assessed using a numeric rating scale (NRS), ranging from 0 to 10, where 10 indicates high motivation.

eHealth literacy was assessed using the eHealth Literacy Scale (eHEALS), which comprises 8 items scored on a scale from 1 to 5. The total score ranges from 8 to 40, with a higher score indicating greater health literacy [52].

Self-reported quality of care was assessed using a modified version of the Osteoarthritis Quality Indicator questionnaire [53], adapted for hand OA. This questionnaire consists of 12 items and calculates a pass rate ranging from 0 to 100, with 100 representing the best quality of care.

Pain and stiffness were assessed using an NRS ranging from 0 to 10, where 0 indicates no pain or stiffness. The activity performance of the hand was evaluated with the Measure of Activity Performance of the Hand (MAP-Hand) questionnaire, consisting of 18 items. The scores were averaged to yield a result between 1 and 4, with 1 indicating no problems [54].

Grip strength was measured as the mean of 2 maximal attempts for each hand (in kg) using a Jamar dynamometer [55].

Usefulness and satisfaction with the intervention were measured using an NRS ranging from 0 to 10 (10=best score). The usability of the app was assessed with the System Usability Scale (SUS), which consists of 10 items [56] and is scored on a scale from 0 to 100. Scores above 71 indicate good usability, above 85 indicate excellent usability, and above 91 indicate the best imaginable usability [57].

Data on adherence to the intervention, including both exercise and informative videos, were collected from the app. Participants logged each exercise session within the app, while adherence to the informative videos was recorded only when participants watched all weekly videos and completed the associated weekly quiz. High adherence to the exercise program was defined as completing at least two exercise sessions per week for a minimum of 8 weeks, while high adherence to the informative videos was defined as watching at least two-thirds of the videos.

Trial logistics were deemed feasible if at least 80% of participants attended the follow-up assessment and had used the app at least once (ie, watched 1 video) during the intervention period. Additionally, the overall trial logistics needed to be considered acceptable by the study group.

Based on previous research on first-line treatment for hand OA [42,43,58], we considered the clinical outcomes of the intervention feasible if significant improvements were observed in pain, stiffness, grip strength, activity performance, and self-reported quality of care.

The technical usability of the app was deemed feasible with a mean SUS score of ≥71 and mean usefulness and satisfaction scores of ≥7. Additionally, the overall experiences of using the app, as reported in focus group interviews, were considered acceptable by the study group.

### Data Collection

All data, including baseline and follow-up questionnaires, app data, and voice recordings from the focus group interviews, were collected through Nettskjema and transmitted as encrypted data to TSD for secure storage.

During the follow-up phase, 5 focus group interviews were conducted with 2-8 participants from various recruitment settings. The semistructured interview guide included questions regarding their experiences with the app; their perceptions of the videos, exercises, reminders, and feedback; and any specific features they appreciated or disliked and would like to change. Participants were also invited to share any content they would like to add or remove, along with other suggestions for improving the app.

### Analyses

#### Quantitative Data

Descriptive statistics are reported as mean and SD for normally distributed data, as median and IQR (25th and 75th percentiles) for nonnormally distributed data, and as frequency and percentage for categorical data. Paired *t* tests (2-tailed) were used to assess changes from baseline to the 3-month follow-up, with a significance level set at *P*<.05. Adherence to exercises and informative videos is presented graphically. No imputation of missing responses was conducted.

#### Qualitative Data

The recordings from the focus group interviews were transcribed verbatim and subsequently anonymized by a research assistant (KAF). The transcripts were then read and analyzed by 2 individuals (KAF and IK), both educated as health care providers (nurse and occupational therapist, respectively) and experienced in conducting qualitative research. A thematic analysis was performed following Braun and Clarke’s [59] approach. This process involved searching for patterns and phrases related to the aims of the focus group interviews. In accordance with this approach, main themes were identified as part of the coding process. We specifically focused on the informants’ opinions and experiences regarding what worked well and what did not in the app, along with their suggestions for changes and improvements.

## Results

### Feasibility Study

A total of 71 participants were included in the feasibility study. Most participants were women, and the mean age was 64 (SD 8) years; 7 of the 71 participants (10%) were recruited by GPs, 30 (42%) from Diakonhjemmet Hospital, and 34 (48%) were collectively recruited from the Norwegian Rheumatism Association and through the daily press. Baseline demographics are shown in Table 1.

Of the 71 participants, 57 (80%) completed the follow-up assessment; 1 withdrew due to hand pain, 3 withdrew due to other medical conditions (stroke/surgery), and 10 did not provide any reason for withdrawal. All measures improved significantly from baseline to follow-up (Table 2).

**Figure 1:**
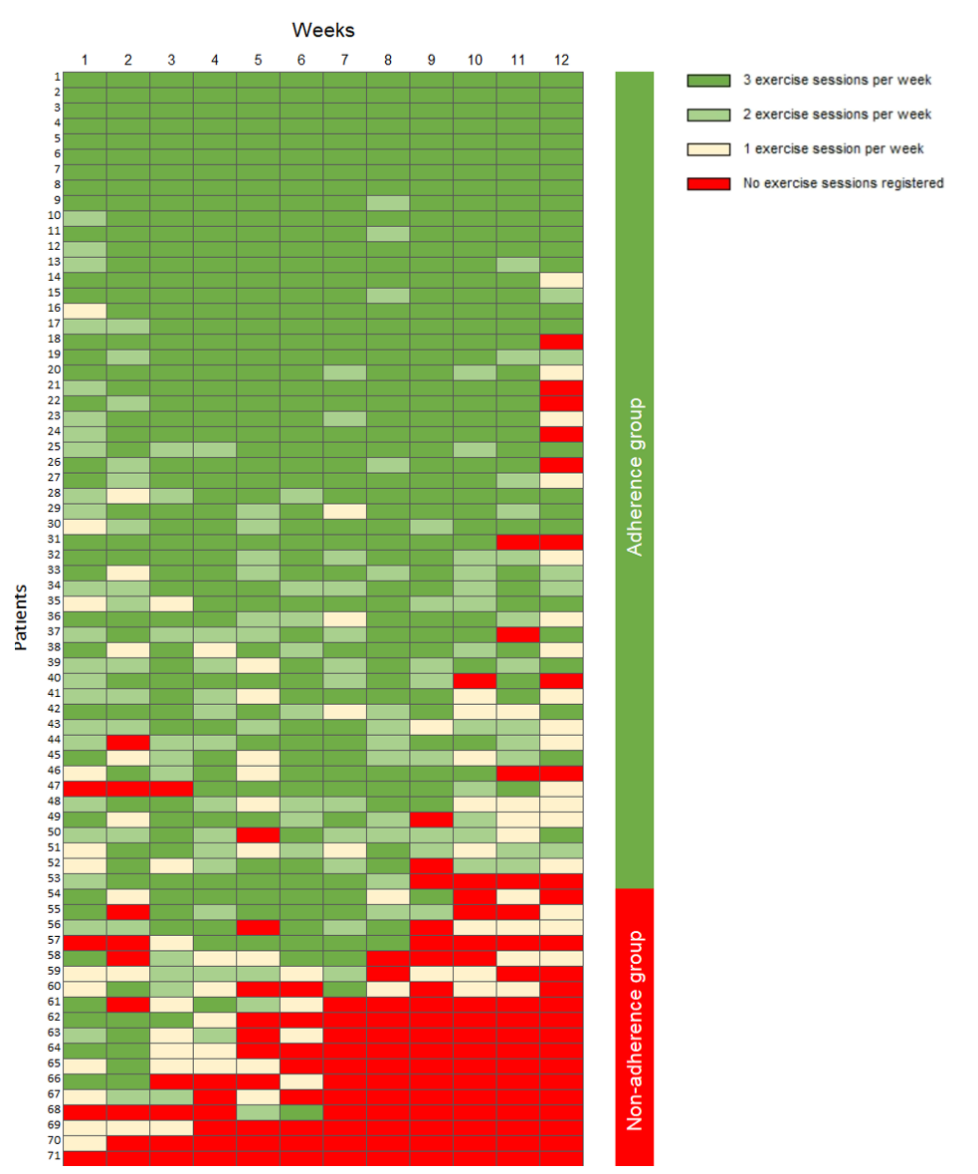
Adherence to the 12-week exercise program in the app-based self-management intervention in participants with hand osteoarthritis included in the feasibility study (n=71). Each row represents an individual participant, and each column represents the individual week of the intervention.

All but 1 participant used the app at least once during the intervention period. A total of 53 of the 71 participants (75%) adhered to the exercise program (Figure 1), with a median number of exercise sessions completed of 29 (IQR 22-33). Additionally, 36 of the 71 (51%) participants adhered to at least two-thirds of the informational videos. The usability of the app was regarded as “best imaginable,” with a mean SUS score of 91.5 (SD 9.2). Participants’ motivation for continued exercise remained high even after the intervention (median 8, IQR 7-10). They were highly satisfied with the app (median 8, IQR 7-10) and considered the intervention to be very useful (median 9, IQR 7-10).

For those who answered all monthly questionnaires in the app (47/71 participants), we found significant improvements in stiffness, pain at rest, pain during activity, and disease activity from baseline to 1 month. These changes were maintained at the following time points (2 and 3 months; Multimedia Appendices 1-3).

**Table 1:** Descriptive statistics of participants with hand osteoarthritis who were included in the feasibility study (n=71).

Descriptive statistics		Values
Females, n (%)		61 (86)
Age (years), mean (SD)		64 (8)
BMI, median (IQR)		24.2 (22.5-27.4)
Living together with someone, n (%)		54 (76)
**Work status, n (%)**		
	Working (full time or part-time)	32 (45)
	Sick leave	2 (3)
	Retired	28 (39)
	Disability pension	8 (11)
	Unemployed	1 (1)
Higher level of education (bachelor and above), n (%)		62 (87)
**Symptomatic hand, n (%)**		
	Right	25 (35)
	Left	14 (20)
	Both	32 (45)
Number of painful joints (0-30), median (IQR)		9 (4-16)
eHealth literacy (eHEALS^a^, 8-40, 40=high health literacy), mean (SD)		29.3 (4.4)
Motivation for exercising (NRS^b^ 0-10, 10=highest motivation), mean (SD)		9 (8-10)

^a^eHEALS: eHealth Literacy Scale.

^b^NRS: numeric rating scale.

**Table 2:** Change in self-reported quality of care and clinical outcomes in participants with hand OAa at baseline and 3-month follow-up in the feasibility study, analyzed with paired sample t test (2-tailed) and reported as mean (SD), mean difference (95% CI), and P-value.

Outcomes			n		Baseline (SD)		Follow-up (SD)		Difference (95% CI)		Paired sample *t* test *P* value
**Self-reported quality of care**											
	Hand OA quality indicators (OA-QI^b^, scored 0-100, 100=best quality)	56		43.2 (25.9)		79.6 (20.4)		36.4 (29.7 to 43.1)		<.001	
**Clinical outcomes**											
	Grip strength right hand (mean of 2 reps, kg)	57		23.5 (9.5)		26.4 (9.0)		2.9 (1.7 to 4.1)		<.001	
	Grip strength left hand (mean of 2 reps, kg)	57		22.2 (8.9)		25.4 (8.2)		3.2 (1.9 to 4.6)		<.001	
	Activity performance (MAP-Hand^c^, scored 1-4, 1=no problems)	56		1.76 (0.42)		1.58 (0.43)		–0.18 (–0.11 to –0.25)		<.001	
	Pain at rest (NRS^d^, scored 0-10, 0=no pain)	55		4.3 (2.6)		3.1 (2.2)		–1.2 (–0.5 to –1.8)		.001	
	Pain in activity (NRS, scored 0-10, 0=no pain)	55		5.2 (2.1)		3.5 (2.9)		–1.7 (–1.2 to –2.2)		<.001	
	Stiffness (NRS, scored 0-10, 0=no stiffness)	56		5.4 (2.0)		3.6 (2.2)		–1.9 (–1.3 to –2.4)		.001	

^a^OA: osteoarthritis.

^b^OA-QI: Osteoarthritis Quality Indicator questionnaire.

^c^MAP-Hand: Measure of Activity Performance of the Hand.

^d^NRS: numeric rating scale.

### Focus Group Interviews

#### Themes

A total of 21 participants engaged in the 5 focus group interviews, with 15 of them being women. Excerpts from the focus group transcripts were categorized into 3 themes: “experiences with the use of the app,” “the content of the intervention,” and “improvements to the app.” Subthemes related to “the content of the intervention” included informative videos, feedback, quizzes, and hand exercises, while subthemes related to “improvements to the app” concerned possible changes to the current version and perspectives on potential extensions for a new version.

#### Experiences With the Use of the App

The results from the focus groups showed that, in general, the participants appreciated the app. They felt that the app contained a comprehensive package of relevant exercises while also providing information about the disease and access to peer experiences. This reduced their need to seek weekly health care services (ie, physiotherapy) and gave them hope that there was something they could do to improve their situation. One participant summarized the experience as follows:

It was kind of a fun tool, because I was at the very beginning [of the disease course] and sort of understood that this is a progressive disease. And it gave me hope; ‘oh, there's something here that can help me’. So it was...I liked it very much, it [the app] really means something.

female, 66 years

The majority found it easy to navigate the app. One participant stated:

I actually found it relatively easy to use. I easily get freaked out by technical stuff like this, but even I managed to make it work.

female, 69 years

In their busy everyday lives, participants mentioned that it could be challenging to remember to exercise regularly. Some also found the exercises quite boring, which made it easier to forget about them. Therefore, many participants highly valued the reminders.

When asked if there was any particular part of the app they appreciated, most participants cited the informative videos and the exercise program. One participant stated:

I thought it was extremely informative with these little videos and information. And knowing that even if it hurts, it’s not dangerous, you can just keep going. That gives kind of a security. Yeah, I thought it was really good.

female, 66 years

#### The Content of the Intervention

The majority found it easy to understand the content of the informative videos, exercise videos, and questionnaires.

Regarding the feedback provided in the app—such as summaries and graphs visualizing levels of pain, stiffness, activity performance, the number of exercise sessions, feedback messages, and awards—participants’ opinions were divided. Some found the feedback (eg, receiving awards) unnecessary or even childish, while others thought the various forms of feedback were encouraging. One participant stated:

I felt a bit guilty when I only got bronze, right? I thought, now I need to sharpen up. I want to get gold and silver again.

female, 60 years

The same was true for the quizzes. While some thought the questions were (too) easy, others found them difficult. One participant explained as follows:

When I started, I thought; O my God, do I really have to do this, I can’t remember all these things. But then I realized that I can. There are only five or six questions, and I did it. Sometimes I had to repeat the quiz three times to get it right. It helps.

male, 76 years

Regarding the exercise program, participants generally appreciated it. Many commented that the program’s progression from relatively simple to more complex or intensive exercises was beneficial. They found it manageable in terms of time commitment, appreciated the flexibility the app offered in deciding when to exercise, and valued the reminders it provided. Additionally, participants liked that they could change the day and time for exercising throughout the 3-month period. Some also mentioned that after a while, they knew the exercises by heart and started doing them anywhere. One participant stated:

I could sit and do it on the bus on the way to work...Kind of like doing everyday exercises without you having to sit and watch [the videos].

male, 56 years

#### Improvements to the App

When asked about changes that would improve the app, participants identified several technical bugs that needed to be fixed. Regarding the exercises, general suggestions included more clearly explaining the aims of the different hand exercises and emphasizing that hand exercises relieve symptoms. Participants also suggested including a greater variety of effective exercises, adding a “library” of different exercises to choose from, and accompanying the videos with music to make them more enjoyable and help with timing.

Suggestions related to specific exercises included providing a more detailed description of the stability exercises, simplifying the coordination exercise, and changing the stretching duration from 30 to 15 seconds. They further requested more practical advice on managing hand OA in everyday life, additional videos featuring work activities such as using a screwdriver or other tools, information on where to purchase the assistive devices demonstrated in the videos, and a video explaining the necessary equipment for performing the hand exercises. One participant suggested making the videos more entertaining by using animations of a humorous character demonstrating the exercises, while another expressed a desire for more detailed information about medication. A third participant suggested that the app begin with a motivational video featuring individuals who have completed the program sharing their experiences. Another user recommended including a video on how to navigate the app.

Some participants commented that hand pain and stiffness could vary between hands, making it challenging to summarize the ratings of both hands into a single value. Many also found the concept of “stiffness” difficult to understand and suggested using the term “joint mobility” instead.

Several participants expressed a desire for an extension of the app that would allow them to continue receiving reminders even after the 3-month intervention period.

#### Changes to the App in Response to Feedback From Participants in the Feasibility Study

Based on the results from the feasibility study, we made several changes to the app. Technical errors were fixed by the software developers. Regarding the assessments, the ratings of hand pain and stiffness were changed from an overall rating to separate ratings for the right and left hands, respectively. Additionally, the term “stiffness” was changed to “joint mobility,” which is more in line with the aim of the exercise program. Based on the focus group interviews, we added an animated video explaining how to use the app, a motivational video featuring 2 users sharing their positive experiences with the app, a video demonstrating the exercise equipment used in the app, and a video showcasing assistive devices and adaptations of tools commonly used by men. Two of the exercise videos were also slightly revised. To obtain a more accurate measure of adherence to the informative videos, registration of which videos are viewed is now conducted independently of whether the participant answers the quizzes. Additionally, we allowed for continued use of the app after the 3-month intervention period.

## Discussion

### Principal Findings

In this study, we used the Medical Research Council framework for the development and evaluation of complex interventions, along with previous research, PRP and user input, Social Cognitive Theory, the BCT Taxonomy, and health literacy principles to guide the development and feasibility testing of an app (Happy Hands), a self-management intervention for people with hand OA. Our development process involved a multidisciplinary team of PRPs, clinicians, and researchers. Additionally, we combined quantitative and qualitative methods to develop, assess, and gather feedback on the app’s design, content, and outcomes. We believe that this meticulous process resulted in the app more effectively meeting users’ requirements for information, feedback, encouragement, flexibility, and follow-up, thereby potentially leading to sustainable and lasting behavior changes. This assumption is supported by the results of the feasibility study, which show an increase in self-reported quality of care; significant and positive changes in pain, stiffness, grip strength, and activity performance of the hand; high adherence to watching the informative and exercise videos; and a high level of satisfaction with the app.

In line with international recommendations for the inclusion of patient representatives in scientific studies [60], PRPs have been involved from the very beginning in the development of the app. Their contributions were crucial in selecting relevant themes, designing the app, ensuring the quality of the content, making the information easily understandable, and enhancing trustworthiness and relevance by sharing their experiences in short videos included in the app. They have also participated in discussing and reporting the results. Furthermore, we gathered the experiences of those who participated in the feasibility testing and used their feedback to adjust the app before testing it in a randomized controlled trial. As reflected in the focus group interviews, we believe this has contributed to the app having relevant content delivered through a user-friendly interface, thereby enhancing empowerment and helping users improve their function and self-management.

All the needs and requirements expressed by the PRPs during the development phase were operationalized into app elements and BCTs, with the exception of their desire for social and professional support. Incorporating a chat function to facilitate such support would violate restrictions on storing sensitive health information on mobile devices. Furthermore, because the app was designed to function both as a standalone intervention and as a supplement to usual treatment, the option to receive advice and supervision from a health care professional was not included. However, the app incorporated several videos featuring advice on how to manage the disease from individuals who themselves have hand OA. These videos were intentionally included to offer users a form of social support.

In the development of the app, we utilized the BCT Taxonomy to incorporate a broad range of action mechanisms that guide and motivate users to implement health-promoting strategies. Additionally, it facilitated the examination of possible mechanisms of action and enabled us to review and enhance the app’s efficacy [61]. In line with the key aspects emphasized by the PRPs, we integrated a variety of BCT components designed to provide positive feedback and rewards to participants, while avoiding threats or negative feedback. The focus group interviews revealed differing opinions among participants regarding these components. Despite this variation, most participants appreciated at least one of the available elements.

After we began developing our app, 2 other apps designed for people with RA were introduced [62,63]. The content of our app aligns well with these 2, as all 3 include hand exercises and provide feedback on progress based on self-reported pain and function. Similar to the Happy Hands app, the MarHand therapy app developed by Tonga et al [62] also provides instructions on how to use the app, allows users to choose when to exercise, and includes reminders and feedback on exercise adherence. By contrast, the CareHand app offers patient education but lacks reminders or motivational feedback within the app [63]. In a recent study, the CareHand app was adapted for people with hand OA [64] and tested in randomized controlled trials with participants who had either RA or hand OA [63,64]. Both trials demonstrated that the app was superior to usual care in improving hand function and pain, and for people with RA, it also improved work performance [63,64]. However, these results should be interpreted cautiously due to small sample sizes and a lack of efficacy on secondary outcomes for people with RA. Meanwhile, the MarHand therapy app developed by Tonga et al has not yet been tested for its effect or cost-effectiveness.

Exercise adherence has been shown to significantly increase the probability of treatment response—such as improvements in pain, function, and/or disease burden—in patients with hand OA [65]. The findings in this study demonstrate similar levels of exercise adherence and comparable improvements in pain, grip strength, and activity performance when compared with a previous study that evaluated a 3-month self-management intervention for people with thumb base OA [58]. In that previous study, participants were provided with assistive devices, orthoses, and instruction on hand exercises at baseline, along with additional support from an occupational therapist after 14 days [58]. The comparable exercise adherence observed in our study suggests that participants were able to effectively engage in the prescribed exercises without the need for direct supervision or ongoing assistance from a health care professional.

Based on the results from the feasibility study, we have now moved to the third phase of the framework for developing and evaluating complex interventions. We are currently recruiting participants with hand OA for a large-scale randomized controlled trial to assess the (cost-) effectiveness of the app. Ultimately, our goal is to make the app available to everyone as a standalone self-management intervention, allowing us to deliver high-quality hand OA care to people in both urban and rural areas of Norway.

This study has several strengths and limitations. We aimed to develop a user-friendly app that complies with GDPR, ensuring that all data collected through the app is encrypted and sent directly to a secure server. We also consider it a strength that we followed the framework for complex interventions, incorporated health literacy principles, and conducted a thorough feasibility study with participants recruited from primary and specialist health care settings, a patient organization, and through mass media. However, the low number of participants recruited from GPs can be seen as a limitation. A limitation is that access to the app requires users to have a smartphone, basic technological knowledge, and proficiency in Norwegian. Additionally, some participants were unable to download the app due to having older smartphones. We also recognize that our participants were highly motivated to exercise and had a high level of education and health literacy, which may limit the generalizability of the results to those who are less motivated or have lower levels of education or health literacy. Although we focused on accessibility throughout the development of the app, we did not systematically adhere to the Web Content Accessibility Guidelines 2.0 [66]. Future development studies should comply with these guidelines. While the feasibility study demonstrates promising results, including significant improvements in pain, stiffness, grip strength, and activity performance, it does not include a control group and experienced a degree of dropout. Therefore, our results should be interpreted with caution. Future studies should also recruit individuals with lower levels of motivation, education, and health literacy to gain a more comprehensive understanding of app usage.

### Conclusions

The Happy Hands app was deemed highly usable and beneficial by the participants. The results from this feasibility study also suggested that the app may enhance the quality of care, grip strength, activity performance, and reduce pain and stiffness. However, definitive conclusions must be established through a randomized controlled trial.

## Appendix

Multimedia Appendix 1 Change in stiffness in the hands across the 12-week intervention period in people with hand osteoarthritis included in the feasibility study (n=47). Stiffness was reported on a monthly basis, including baseline on a numeric rating scale (0-10, 0=no stiffness).

Multimedia Appendix 2 Change in pain at rest in the hands across the 12-week intervention period in people with hand osteoarthritis included in the feasibility study (n=47). Pain at rest was reported on a monthly basis, including baseline on a numeric rating scale (0-10, 0=no pain).

Multimedia Appendix 3 Change in pain in activity in the hands across the 12-week intervention period in people with hand osteoarthritis included in the feasibility study (n=47). Pain in activity was reported on a monthly basis, including baseline on a numeric rating scale (0-10, 0=no pain).

## Abbreviations

BCT: behavior change technique
eHEALS: eHealth Literary Scale
EULAR: European Alliance of Associations for Rheumatology
GDPR: General Data Protection Regulation
GP: general practitioner
MAP-Hand: Measure of Activity Performance of the Hand
NRS: numeric rating scale
OA: osteoarthritis
PRP: patient research partner
RA: rheumatoid arthritis
SUS: System Usability Scale
TSD: Services for Sensitive Data
USIT: The University Information Technology Center

## Data Availability

The data set generated and analyzed during this study is not publicly available due to General Data Protection Regulation (GDPR)/local regulations at Diakonhjemmet Hospital. Access to a Norwegian trial version of the Happy Hands app can be provided upon reasonable request to the corresponding author (ATT), depending on the availability to download the app from countries outside of Norway.
