# Supplementary figures and images for: Development of the Happy Hands Self-Management App for People with Hand Osteoarthritis: Feasibility Study
Source: JMIR Form Res. 2024 Oct 29;8:e59016. doi: 10.2196/59016 (PMC11558211; doi:10.2196/59016)

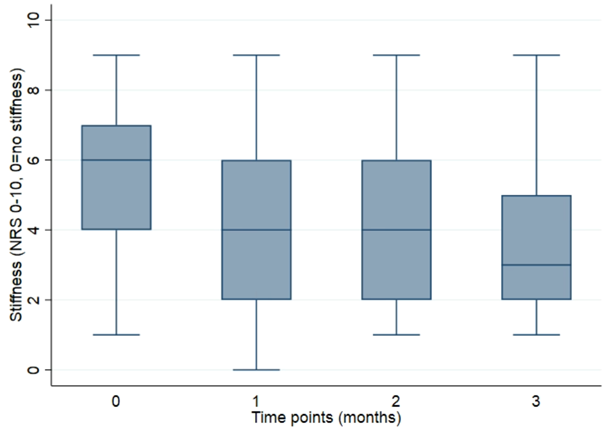

Supplement: Multimedia Appendix 1 [file formative_v8i1e59016_app1.png]

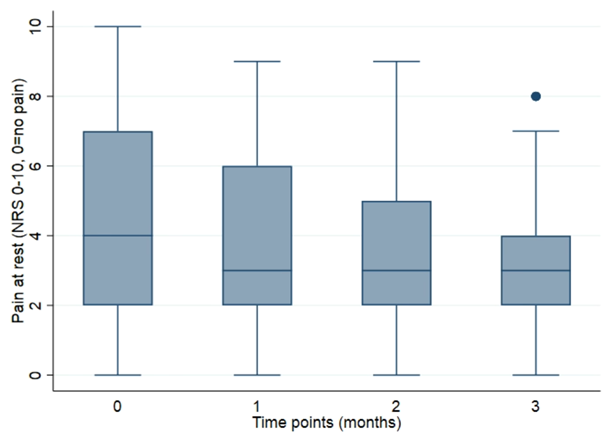

Supplement: Multimedia Appendix 2 [file formative_v8i1e59016_app2.png]

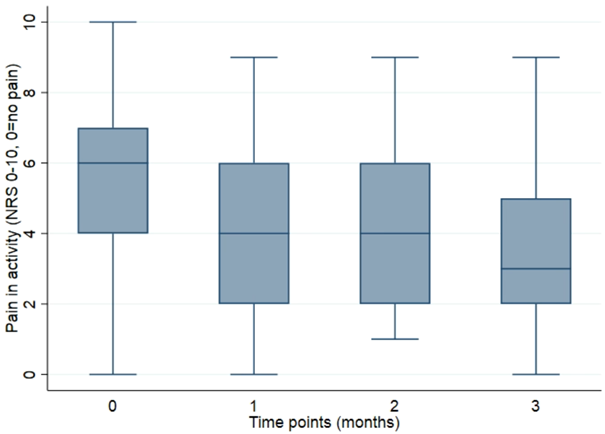

Supplement: Multimedia Appendix 3 [file formative_v8i1e59016_app3.png]
